# Genome sequence of the New Zealand cheese isolate and candidate probiotic strain *Lactiplantibacillus plantarum* FNZ042

**DOI:** 10.1128/mra.00647-24

**Published:** 2024-09-30

**Authors:** Eduardo L. de Almeida, Paul W. O’Toole, Shalome A. Bassett

**Affiliations:** 1 Fonterra Microbiome Research Centre, University College Cork, Cork, Ireland; 2 APC Microbiome Ireland, University College Cork, Cork, Ireland; 3 School of Microbiology, University College Cork, Cork, Ireland; 4 Fonterra Research & Development Centre, Palmerston North, New Zealand; 5 Riddet Institute, Massey University, Palmerston North, New Zealand; The University of Arizona, Tucson, Arizona, USA

**Keywords:** genomics, bioinformatics, probiotics, *Lactiplantibacillus plantarum *FNZ042

## Abstract

The complete genome sequence of the candidate probiotic strain *Lactiplantibacillus plantarum* FNZ042 was determined using a hybrid genome assembly comprising data from Illumina and PacBio sequencing platforms. The genome assembly comprised 3,265,637 bp, including the complete circular chromosome and three circular plasmids.

## ANNOUNCEMENT

*Lactiplantibacillus plantarum* FNZ042, a candidate probiotic strain from the Fonterra Culture Collection (Palmerston North, New Zealand) deposited with the Australian Measurement Institute (deposit number V23/018420), was sourced from New Zealand cheddar cheese in 1989. Taxonomy was initially determined using traditional culture methods and 16S rRNA gene sequencing.

A hybrid assembly approach combining Illumina and PacBio reads was employed. The strain was purity-streaked twice from the original −80°C glycerol stock. A single colony was incubated statically overnight (De Man–Rogosa–Sharpe (MRS) broth, 37°C) for master and working glycerol stocks and gDNA isolation for Illumina sequencing. DNA extraction for PacBio sequencing required an additional culturing step where 1 mL overnight culture was added to 12 mL of pre-warmed MRS broth and incubated at 37°C for 5 hours. For Illumina sequencing, >200 ng of total gDNA was extracted using the QIAamp DNA Mini Kit (QIAGEN, Hilden, Germany), a library prepared using the Nextera XT DNA Library Preparation Kit (Illumina, San Diego, CA, USA), and sequenced on the Illumina MiSeq platform. Paired 150-bp reads (2,183,744 total reads) were analyzed and quality-controlled using fastQC v0.11.9 (1) and Trim Galore v0.6.7 (2), with adapters removed and read ends with quality below Q30 trimmed. For PacBio sequencing, >8.5 µg of total gDNA was isolated using the NucleoBond HMW DNA kit (MACHEREY-NAGEL, Düren, Germany), while library preparation and sequencing were performed by Novogene (Singapore) using the Sequel platform. PacBio subreads were quality-filtered and converted to FASTQ format using BamTools 2.5.1 (3), retaining subreads with a minimum length of 1 kb and minimum quality of 0.75. PacBio sequencing statistics were computed with NanoPlot 1.42.0 (4), resulting in 380,915 unfiltered subreads and N_50_ of 7,568 bp.

Contig circularity was determined following genome assembly using the Unicycler pipeline v0.4.8 in the “normal” mode (5). Genome assembly statistics were computed using QUAST v5.2.0 (6), SeqKit v2.5.1 (7), and Bakta v1.9.3 (8). The total genome length was 3,265,637 bp, G + C content 44.54%, N_50_ of 3,207,670 bp, and L_50_ of 1, with 3,034 predicted coding sequences and a final estimated genome coverage of 982-fold. This included the complete circular chromosome (3,207,670 bp) and three circular plasmids; pFNZ042-01 (46,689 bp), pFNZ042-02 (9,254 bp), and pFNZ042-03 (2,024 bp). Taxonomy was confirmed using the GTDB-Tk pipeline v2.3.2 (9). The public version of the assembly was annotated using PGAP (10), and all analyses were based on local annotation using Bakta.

No antimicrobial resistance (AMR) or virulence genes were detected, as defined by the European Food Safety Authority (EFSA) recommendations (11), using the AMRFinderPlus pipeline v3.12.8 (12) and ResFinder v4.6.0 (13) at 80% sequence identity and 70% coverage of the reference sequence. Likewise, no known genes involved in biogenic amine synthesis were detected, as evaluated using KofamScan v1.3.0 (14–17). *In vitro* safety assessment was performed according to EFSA guidelines (18) using the ISO 10932|IDF 223:2010 standards for determining the minimal inhibitory concentration (MIC) of antibiotics in lactic acid bacteria (19). All MIC values were lower or equal to the EFSA cutoffs, except for tetracycline and chloramphenicol (Table 1A). However, a previous analysis of 10 *L*. *plantarum* strains showed that most were resistant to these antibiotics (20), and a recent survey suggests that a higher MIC cutoff should be used for *L. plantarum* to better differentiate between susceptible strains and those with acquired resistance (21). This could indicate intrinsic resistance in the species and therefore minimal potential for horizontal transfer. This is supported by the absence of these AMR genes in FNZ042. D-/L-lactate production was assessed using the Megazyme D-/L-Lactic Acid (Rapid) Assay Kit (Neogen, Bray, Ireland) following the manufacturer’s instructions, as 60:40 wt/vol (Table 1B), consistent with other *L. plantarum* probiotic strains (22). The high-quality genomic data generated for *L. plantarum* FNZ042 in this study, together with these safety assessments, will assist in the evaluation of this strain as a potential probiotic.

**TABLE 1:** *In vitro* safety assessment for L. plantarum FNZ042 (A) MIC results (B) D-/L-lactate production ratio results

(A) MIC results (µg/mL) – noting that as per EFSA guidelines, evaluation of resistance to vancomycin, streptomycin, and ciprofloxacin is not required for *L. plantarum* strains										
	Ampicillin	Gentamycin		Kanamycin		Erythromycin	Clindamycin		Tetracycline	Chloramphenicol
*L. plantarum* FNZ042	2	<1		8		0.25	0.5		64	16
EFSA guidelines for *L. plantarum*	2	16		64		1	4		32	8

(B) D-/L-lactate production ratio results										
D-Lactate concentration (g/L)	L-Lactate concentration (g/L)		D-/L-Lactate Ratio		Lactate total (g/L)		D-Lactate %	L-Lactate %		Growth conditions
10.605	6.949		1.526		17.554		60.4%	39.6%		MRS broth, aerobic, 37°C, 24 h

## Data Availability

This Whole Genome Shotgun project has been deposited in GenBank under the accession number GCA_040356725.1. Sequencing reads have been deposited in the Sequence Read Archive under accession numbers SRX24875441 (Illumina) and SRX24875442 (PacBio).
